# Impact of the COVID-19 pandemic on cardiovascular mortality and catherization activity during the lockdown in central Germany: an observational study

**DOI:** 10.1007/s00392-020-01780-0

**Published:** 2020-11-21

**Authors:** Holger M. Nef, Albrecht Elsässer, Helge Möllmann, Mohammed Abdel-Hadi, Timm Bauer, Martin Brück, Holger Eggebrecht, Joachim R. Ehrlich, Markus W. Ferrari, Stephan Fichtlscherer, Ulrich Hink, Hans Hölschermann, Rifat Kacapor, Oliver Koeth, Serguei Korboukov, Steffen Lamparter, Alexander J. Laspoulas, Ralf Lehmann, Christoph Liebetrau, Tobias Plücker, Jörn Pons-Kühnemann, Volker Schächinger, Bernhard Schieffer, Peter Schott, Matthias Schulze, Claudius Teupe, Mariuca Vasa-Nicotera, Michael Weber, Christoph Weinbrenner, Gerald Werner, Christian W. Hamm, Oliver Dörr, for the CoVCAD –Study Group, Abdulaziz Al-Hariri, Ursula Boening, Sven Faßbender, Charlotte Funk, Moritz Haas, Catharina Hamm, Felix Hofmann, Konstantinos Karatolios, Tore Körschgen, Kerstin Michalek, Shari Schauberger, Sylvana Neumann, Thorsten Runde, Wiebke Rutsatz, Michael Stanisch, Peter Schifferings, Eberhard Schneider, Maren Weferling

**Affiliations:** 1 Department of Cardiology, Justus Liebig University Giessen, University Hospital Giessen, Medical Clinic I, Klinikstrasse 33, 35392 Giessen, Germany; 2 Department of Cardiology, Herz-Kreislauf-Zentrum Klinikum Hersfeld-Rotenburg, Rotenburg, Germany; 3 Department of Cardiology, Klinikum Oldenburg, Oldenburg, Germany; 4 St.-Johannes-Hospital Klinik Für Innere Medizin I, Dortmund, Germany; 5 Department of Cardiology, DRK Kliniken Nordhessen, Kassel, Germany; 6 Department of Cardiology, Sana Klinikum Offenbach, Offenbach, Germany; 7 Department of Cardiology, Lahn-Dill-Kliniken, Klinikum Wetzlar, Wetzlar, Germany; 8 Department of Cardiology, Agaplesion Frankfurter Diakonie Kliniken, Frankfurt, Germany; 9 Department of Cardiology, St. Josefs-Hospital Wiesbaden, Wiesbaden, Germany; 10 Department of Cardiology, Helios Dr. Horst Schmidt Kliniken, Wiesbaden, Germany; 11 Department of Cardiology, Klinikum Frankfurt Höchst, Frankfurt am Main, Germany; 12 Department of Cardiology, Hochtaunus-Kliniken, Bad Homburg, Germany; 13 Department of Cardiology, Kliniken Des Main-Taunus-Kreises, Bad Soden am Taunus, Germany; 14 Department of Cardiology, GPR Gesundheits- Und Pflegezentrum Rüsselsheim, Rüsselsheim, Germany; 15 Hessenklinik Stadtkrankenhaus Korbach, Korbach, Germany; 16 Department of Cardiology, Diakonie-Krankenhaus Wehrda, Marburg, Germany; 17 Department of Cardiology, Asklepios Kliniken Langen, Langen, Germany; 18 Department of Cardiology, Kerckhoff Heart Center, Bad Nauheim, Germany; 19 Department of Cardiology, Eichhof-Stiftung Lauterbach, Lauterbach, Germany; 20 Justus Liebig University Giessen, Medical Statistics, Institute of Medical Informatics, Giessen, Germany; 21 Department of Cardiology, Klinikum Fulda, Fulda, Germany; 22 Department of Internal Medicine/Cardiology and Angiology, University Hospital of Marburg, Marburg, Germany; 23 Department of Cardiology, Klinikum Werra Meissner GmbH, Eschwege, Germany; 24 Department of Cardiology, Asklepios Schwalm-Eder-Kliniken, Schwalmstadt, Germany; 25 Department of Cardiology, Krankenhaus Sachsenhausen, Frankfurt am Main, Germany; 26 Department of Cardiology, University of Frankfurt, Frankfurt, Germany; 27 Department of Cardiology, Kreisklinik Groß-Umstadt, Groß-Umstadt, Germany; 28 Department of Cardiology, Klinikum Hanau, Hanau, Germany; 29 Department of Cardiology, Klinikum Darmstadt, Darmstadt, Germany

**Keywords:** SARS-CoV2 pandemic, COVID-19, Chronic coronary syndrome, Acute coronary syndrome, Cardiovascular mortality

## Abstract

**Aims:**

During the COVID-19 pandemic, hospital admissions for cardiac care have declined. However, effects on mortality are unclear. Thus, we sought to evaluate the impact of the lockdown period in central Germany on overall and cardiovascular deaths. Simultaneously we looked at catheterization activities in the same region.

**Methods and results:**

Data from 22 of 24 public health-authorities in central Germany were aggregated during the pandemic related lockdown period and compared to the same time period in 2019. Information on the total number of deaths and causes of death, including cardiovascular mortality, were collected. Additionally, we compared rates of hospitalization (*n* = 5178) for chronic coronary syndrome (CCS), acute coronary syndrome (ACS), and out of hospital cardiac arrest (OHCA) in 26 hospitals in this area. Data on 5,984 deaths occurring between March 23, 2020 and April 26, 2020 were evaluated. In comparison to the reference non-pandemic period in 2019 (deaths: *n* = 5832), there was a non-significant increase in all-cause mortality of 2.6% [incidence rate ratio (IRR) 1.03, 95% confidence interval (CI) 0.99–1.06; *p* = 0.16]. Cardiovascular and cardiac mortality increased significantly by 7.6% (IRR 1.08, 95%-CI 1.01–1.14; *p* = 0.02) and by 11.8% (IRR 1.12, 95%-CI 1.05–1.19; *p* < 0.001), respectively. During the same period, our data revealed a drop in cardiac catherization procedures.

**Conclusion:**

During the COVID-19-related lockdown a significant increase in cardiovascular mortality was observed in central Germany, whereas catherization activities were reduced. The mechanisms underlying both of these observations should be investigated further in order to better understand the effects of a pandemic-related lockdown and social-distancing restrictions on cardiovascular care and mortality.

**Graphic abstract:**

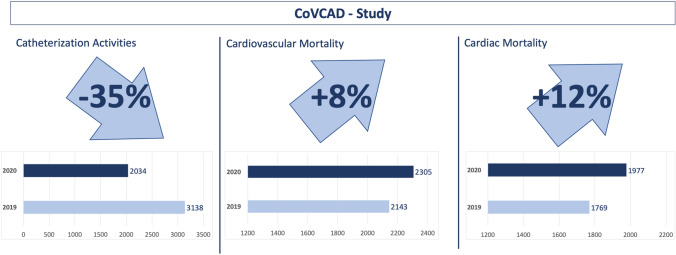

## Introduction

The emergence of the COVID-19 pandemic has affected different spaces in the medical community [1–3]. Countries across Europe have significantly curbed public life in order to halt the spread of the COVID-19 outbreak [1, 2, 4].

In central Germany, the State of Hesse issued a lockdown from March 23rd until April 26th, 2020, ordering approximately 6 million residents to “stay at home”. The official government guidelines stated that people should go shopping “as infrequently as possible”, and leave their homes only for “essentials”. Schools, universities, and all non-essential businesses were closed. Travel within Germany was banned except for health reasons or urgent matters. From several reports coming from US and Europe, it is well known that the epidemiological crisis and the following lockdown period has strongly impacted cardiac care. Analyses demonstrated that in the pandemic the number of visits to ambulatory care practices declined by nearly 60% [5]. Moreover, there was a clear impact of the COVID-19 pandemic on heart failure hospitalization and management [6]. Additionally, the incidence of hospitalization for patients with acute coronary syndromes (ACS) showed a dramatic drop of approximately 40% and was even more pronounced in patients with chronic coronary syndrome with an unknown effect on cardiovascular mortality [7–11].

Against this background, the aim of the present observational CoVCAD (COVid-19 and CArdiovascular Disease) study was to systematically analyze cardiovascular mortality including ACS, heart failure, heart rhythm disorders (summarized as cardiac death), pulmonary embolism, and stroke in central Germany during the lockdown-related “stay at home” reaction in comparison to the same non-pandemic period in 2019 and to investigate changes in numbers of catheterization for cardiac catheterization.

## Methods

### Study design and data acquisition

In a comprehensive analysis, mortality data from local public health authorities in central Germany (Hesse) were aggregated from March 23rd to April 26th, 2020. A total of 22 out of 24 (92%) health authorities from this region participated in the present CoVCAD study. The data were compared to a reference non-pandemic period in 2019 from the same health authorities nationwide for the region of central Germany to ensure a direct comparison between the periods. In addition, information on all-cause mortality and causes of death was gathered by reviewing all available death certificates. Causes of death were documented by a physician at the last medical contact who signed the death certificate, including in-hospital deaths as well as out-of-hospital deaths.

Thereby, we focused on cardiovascular mortality including ACS, heart failure, heart rhythm disorders (summarized as cardiac death), pulmonary embolism, and stroke. Additionally, we assessed mortality resulting from other causes that were summarized as non-cardiovascular death as well as all non-COVID-19 death.

COVID-19 death was documented when the patients were tested positive, irrespective of other comorbidities. During the lockdown, all patients who were admitted to a hospital were tested for COVID-19 if the following criteria of the Robert-Koch-Institute were fulfilled: (1) typical respiratory symptoms, (2) contact with a person who tested positive during the past 14 days, or (3) stay at a region of risk during the past 14 days.

For the analysis of catherization activities, hospitals in central Germany were asked to provide data for all patients who were admitted for cardiac catheterization due to chronic coronary syndromes (CCS), ACS (NSTE-ACS, STEMI), and out-of-hospital cardiopulmonary arrest (OHCA) during the lockdown period from March 23rd to April 26th, 2020. In addition, data were acquired retrospectively from March 23rd to April 26th, 2019 from these catheterization laboratories. A total of 26 hospitals contributed to the present study. Data assessment and participation in the CoVCAD study were optional. For further analysis, patients’ characteristics and procedural data were assessed from the participating hospitals. To exclude variations for catherization activities for other reasons (recent studies, e.g. ISCHEMIA trial; improved primary or secondary prevention) we also analyzed catheter laboratory volumes from January to February 2020 compared with January to February 2019, defined as an immediately adjacent non-pandemic period.

The study protocol was approved by the ethics committee of the medical faculty of the Justus-Liebig-University of Giessen, Germany (AZ 60/20). The investigation conforms to the principles outlined in the Declaration of Helsinki. The statistical analysis was performed by the department of medical statistics at the University of Giessen, Germany.

### Statistical analysis

Categorical variables are reported as numbers and percentages. Comparisons of categorical variables were executed by Pearson χ2-test without continuity correction. Confidence Intervals of relative change of numbers were estimated by Poisson Regression. Continuous variables were evaluated by QQ-Plot and Shapiro–Wilk-Test for normal distribution. As normal distribution was rejected, continuous variables are expressed as median with interquartile range and comparisons were executed by Mann–Whitney test. Incidence rates (Daily Events) were calculated by dividing the number of cumulative admissions by the number of days for each time period (35 days). Incidence rate ratios comparing the 2020 study period with the control period (2019) were estimated by Poisson regression analysis including year as explanation factor and center as confounder. No adjustments for multiple testing were performed. For all statistical analyses, the statistical software R-4.0.0 (R Core Team 2020) was used.

## Results

### Mortality during the pandemic-related lockdown

**Fig. 1:**
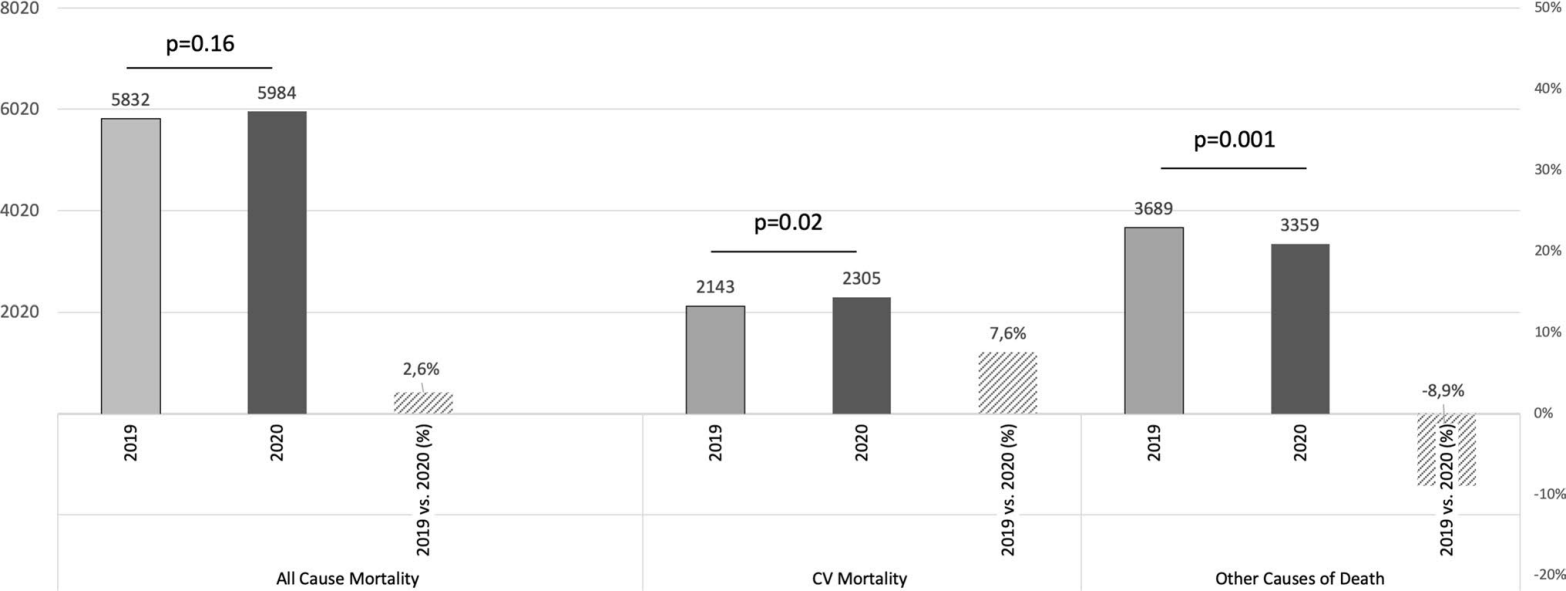
All-cause mortality, cardiovascular mortality, and other causes of death during the lockdown period in 2020 in comparison to the non-pandemic period in 2019

**Fig. 2:**
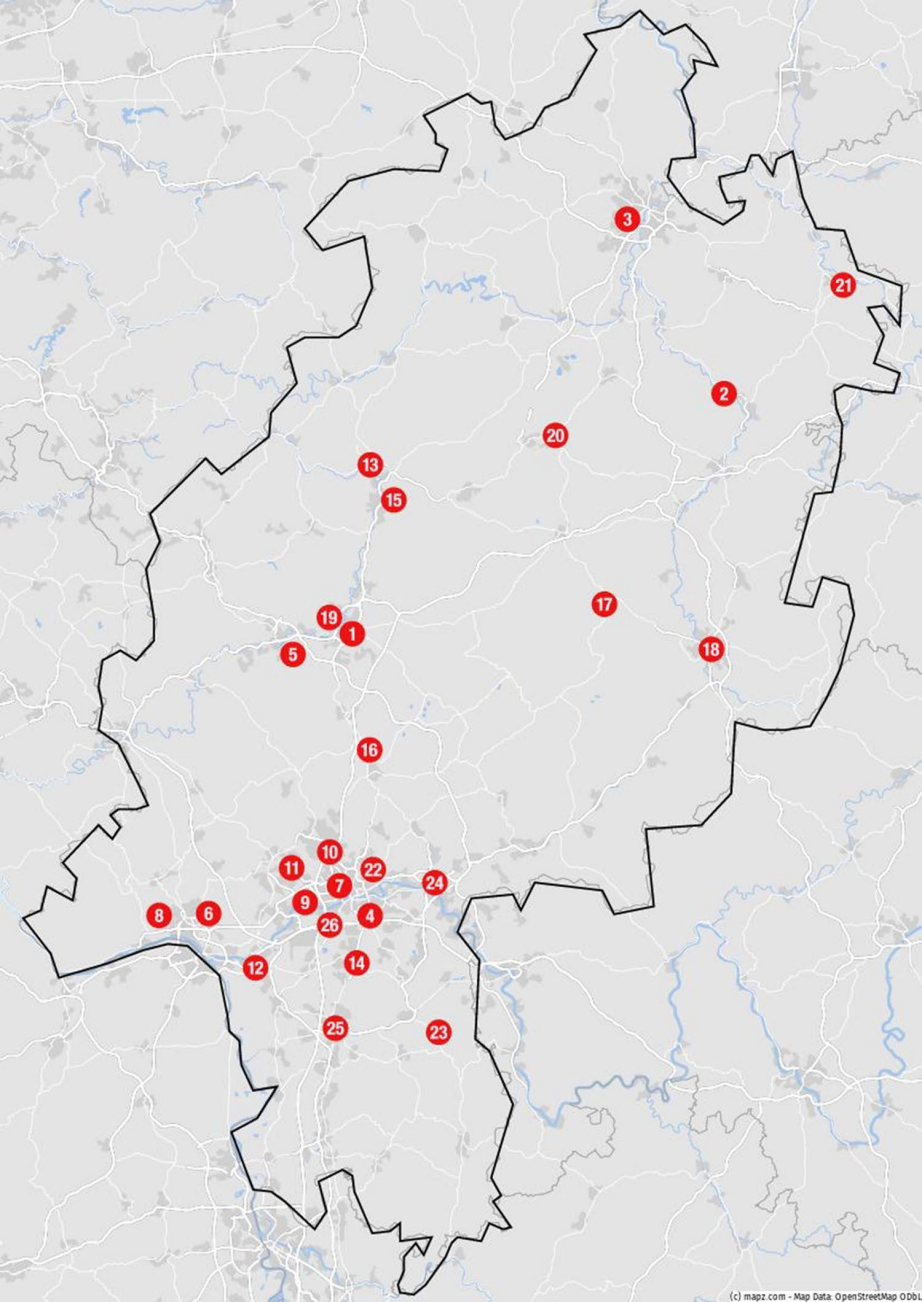
Map of the state of Hesse, including all participating hospitals to the CoVCAD-Study: **(1)** Justus Liebig University Giessen, University Hospital Giessen, Medical Clinic I, Giessen **(2)** Herz-Kreislauf-Zentrum Klinikum Hersfeld-Rotenburg, Dept. of Cardiology, Rotenburg **(3)** Hessenklinik Stadtkrankenhaus Korbach, Korbach **(4)** Sana Klinikum Offenbach, Dept. of Cardiology, Offenbach **(5)** Lahn-Dill-Kliniken, Klinikum Wetzlar, Dept. of Cardiology, Wetzlar **(6)** St. Josefs-Hospital Wiesbaden, Dept. of Cardiology, Wiesbaden **(7)** Agaplesion Frankfurter Diakonie Kliniken, Dept. of Cardiology, Frankfurt **(8)** Helios Dr. Horst Schmidt Kliniken, Dept. of Cardiology, Wiesbaden **(9)** Klinikum Frankfurt Höchst, Dept. of Cardiology, Frankfurt am Main **(10)** Hochtaunus-Kliniken, Bad Homburg, Dept. of Cardiology **(11)** Kliniken des Main-Taunus-Kreises, Dept. of Cardiology **(12)** GPR Gesundheits- und Pflegezentrum Rüsselsheim, Dept. of Cardiology, Rüsselsheim **(13)** Diakonie-Krankenhaus Wehrda, Dept. of Cardiology, Marburg **(14)** Asklepios Kliniken Langen, Dept. of Cardiology, Langen **(15)** Department of Internal Medicine/Cardiology and Angiology, University Hospital of Marburg, Marburg **(16)** Kerckhoff Heart Center, Dept. Of Cardiology, Bad Nauheim **(17)** Eichhof-Stiftung Lauterbach, Dept. of Cardiology **(18)** Klinikum Fulda, Dept. of Cardiology, Fulda **(19)** Agaplesion Evangelisches Krankenhaus Mittelhessen, Dept. of Cardiology Giessen **(20)** Asklepios Schwalm-Eder-Kliniken, Dept. of Cardiology, Schwalmstadt **(21)** Klinikum Werra Meissner GmbH, Dept. of Cardiology, Eschwege **(22)** Krankenhaus Sachsenhausen, Dept. of Cardiology, Frankfurt am Main **(23)** Kreisklinik Groß-Umstadt, Dept. of Cardiology, Groß-Umstadt **(24)** Klinikum Hanau, Dept. of Cardiology, Hanau **(25)** Klinikum Darmstadt, Dept. of Cardiology, Darmstadt **(26)** University of Frankfurt, Dept. of Cardiology, Frankfurt

From March 23rd to April 26th, 2020, a total of 5984 deaths were registered by the participating public health authorities in central Germany (Hesse) during the analyzed lockdown period. In comparison, during the similar non-pandemic period in 2019, the number of deaths was 5,832, resulting in a non-significant increase in all-cause mortality by 2.6% [incidence rate ratio (IRR) 1.03, 95% confidence interval (CI) 0.99–1.03; *p* = 0.02]. This was mainly driven by the absolute number of deaths resulting from COVID-19 (*n *= 320, Table 1; Figs. 1 and 2).

**Table 1:** Mortality data during the lockdown period in 2020 in comparison to the non-pandemic period in 2019

	Events 2019	Events 2020	Delta Events 2019 vs. 2020	Daily Events 2019	Daily Events 2020	Incidence rate ratio (95% CI)	*P*-value
All-cause mortality	5832	5984	152	166.63	170.97	1.03 (0.99–1.06)	0.16
Cardiovascular mortality	2143	2305	162	61.23	65.86	1.08 (1.01–1.14)	0.02
Cardiac mortality	1769	1977	208	50.54	56.49	1.12 (1.05–1.19)	0.001
Stroke	270	213	-57	7.71	6.09	0.79 (0.66–0.94)	0.01
Pulmonary embolism	104	115	11	2.97	3.29	1.11 (0.85–1.44)	0.46
COVID death	–	320	320	–	9.14	–^#^	–
Other	3689	3359	– 330	105.40	95.97	0.91 (0.87–0.95)	0.001

^#^Incidence rate ratio not estimable

During the lockdown period, there was an increase in cardiovascular mortality, comprising cardiac death, pulmonary embolism, and stroke, of 7.6% (IRR 1.08, 95% CI 1.01–1.14; *p* = 0.02) (Fig. 1). Cardiac mortality increased by 11.8% (IRR 1.12, 95% CI 1.05–1.19; *p* < 0.001). The incidence of fatal pulmonary embolism increased by 10.6% (IRR 1.11, 95% CI 0.85–1.44; *p* < 0.46), although this change was without any statistical significance. In contrast, the number of patients who deceased due to stroke (IRR 0.79, 95% CI 0.66–0.94; *p* = 0.01) and other causes of death (IRR 0.91, 95% CI 0.87–0.95; *p* = 0.001) was lower during the lockdown period in 2020 (Table 1).

### Evaluation of cardiac catherization activities

In the final analysis, 5579 patients from 26 hospitals in central Germany (Hesse) were enrolled in the CoVCAD study. The participating hospitals were representative of all hospitals in central Germany, given the fact that there was a balanced proportion of patients enrolled either in university hospitals/heart centers (*n* = 2812) or in district hospitals (*n* = 2767). In addition, the duration of hospital stay did not differ between university hospitals/heart centers (6.7 days) and district hospitals (5.48 days). During the lockdown period from March 23rd to April 26th, 2020, a total of 2034 patients were admitted for cardiac catheterization to the participating hospitals. Out of these patients, 1112 (55%) presented with CCS, 560 (28%) with NSTE-ACS, 300 (15%) with STEMI, and 58 (3%) with OHCA. During the same non-pandemic period in 2019, a total of 3138 patients were admitted, including 2008 (64%) with CCS, 750 (24%) with NSTE-ACS, 311 (10%) with STEMI, and 69 (2%) with OHCA (Table 2).

**Table 2:** Clinical presentation of patients who were admitted for cardiac catheterization during the pandemic-related lockdown period and non-pandemic period

Clinical presentation	2019	2020	Change % (95% CI)^1^	*p *value^1^
CCS, *n*	2008	1112	44.6 (40.4–48.5)	< 0.001
ACS, *n*	1061	860	18.9 (11.3–25.9)	< 0.001
NSTE-ACS, *n*	750	560	25.3 (16.7–33.1)	< 0.001
STEMI, *n*	311	300	3.5 (0–17.7)	0.656
OHCA, *n*	69	58	15.9(0–40.7)	0.330
Procedural characteristics	2019	2020		*p*-value^2^
PCI, *n* (%)	1173/2084 (56)	687/1290 (53)		0.163
LM PCI, *n* (%)	91/1725 (5)	51/1080 (5)		0.516
CTO-PCI, *n* (%)	67/1727 (4)	54/1079 (5)		0.153
Bifurcation-PCI, *n* (%)	79/1724 (5)	47/1080 (4)		0.774
Door to balloon Time (STEMI), median (IQR)	36 (25–52.5)	34 (25–59.8)		0.783^3^

Tests used: ^1^Poisson regression; ^2^Pearson X^2^ test; ^3^Wilcoxon test

*ACS* acute coronary syndrome, *CCS* Chronic coronary syndrome, *LM*: left main, *NSTE-ACS* acute coronary syndrome without ST-elevation, *OHCA* out-of-hospital cardiac arrest, *PCI* percutaneous coronary intervention, *STEMI* ST-elevation myocardial infarction

When comparing the lockdown period 2020 with the reference non-pandemic period in 2019, we found a 44.6% (2008 vs. 1112) decrease in the number of elective procedures, and a drop of 18.9% (1061 vs. 860) in ACS-related procedures was documented. The number of patients was reduced by 25.3% for NSTE-ACS (750 vs. 560) and by 3.5% for STEMI (320 vs. 311) (Table 2). All patient characteristics analyzed are presented in Table 3.

**Table 3:** Demographics of patients undergoing cardiac catherization

Demographics	2019	2020	*P*-value
	*N* = *3545*	*N* = *2034*	
Male, *n* (%)	1852/2826 (66)	1105/1604 (69)	0.023^1^
Age, median (IQR)	70 (60–79)	70 (59–78)	0.052^2^
History of CAD, *n* (%)	940/2034 (46)	555/1178 (47)	0.622^1^
Diabetes mellitus, n (%)	588/1923 (31)	341/1212 (28)	0.145^1^
Hypertension, *n* (%)	1391/1769 (79)	891/1111 (80)	0.313^1^
Currently smoking, *n* (%)	325/1641 (20)	254/1102 (23)	0.041^1^
Chronic kidney disease, *n* (%)	347/1766 (20)	217/1106 (20)	0.985^1^
BMI, median (IQR)	27 (24.6–31)	27.2 (24.2–30.5)	0.321^2^
LV function, median (IQR)	55 (45–60)	55 (45–60)	0.188^2^
ACE-I / ARB, *n* (%)	1124/1740 (65)	753/1089 (69)	0.013^1^
ß-Blocker, *n* (%)	1031/1740 (59)	700/1089 (64)	0.008^1^
Diuretics, *n* (%)	726/1739 (42)	435/1089 (40)	0.343^1^

Tests used: ^1^Pearson χ^2^-test; ^2^Wilcoxon test

Overall, the total number of percutaneous coronary interventions (PCI) was lower in the lockdown period in comparison to the non-pandemic period, although the difference in the relative number of PCI procedures was not statistically significant (Table 2). The relative number of complex PCI procedures also did not differ (CTO: *p* = 0.153, bifurcation: *p* = 0.774, left main: *p* = 0.516). Specific procedural characteristics are presented in Table 2. Catherization activities from the high-volume centers in central Germany showed a declining, but not significant, trend during the immediately adjacent pre-pandemic (January–February 2020) period when compared with 2019 (1958 vs. 1859).

During the COVID-19 pandemic, in-hospital mortality in patients admitted for cardiac catherization was higher when compared with 2019 (58/1,801 vs. 55/3,030, *p* = 0.002). However, the length of hospital stay (6.2 ± 5.9 days vs. 6.7 ± 7.8 days; *p* = 0.657) did not differ when comparing the two periods.

## Discussion

COVID-19 pandemic has placed an enormous strain on the healthcare systems worldwide, with dramatic implications for medical practice [4, 12, 13]. The present pandemic has led to modifications of standard practice in cardiac care, including for patients presenting with acute coronary syndromes [14–16].

Recent data from the USA, China, Spain, and Italy, and also preliminary data from Germany have provided information that the COVID-19 pandemic period has led to a significant reduction in the number of procedures being carried out in interventional cardiology [9, 17–19].

Accordingly, coronary catherization in patients with ACS declined by approximately 40%, and this decline was even more pronounced in patients with CCS [15, 18]. In addition, management of PCI in patients with NSTE-ACS and STEMI was more challenging due to delays in transfer times, prolonged emergency department evaluations, and infection control requirements in catherization laboratories that resulted in delays in treatment [9].

In the central German State of Hesse, with a population of approximately 6 million, the rate of all-cause mortality increased during the COVID-19-related lockdown compared with the same reference period in 2019. More important, the proportion of cardiovascular deaths increased by 7.6%. In accordance with recent reports, in the present study the number of patients with CCS, NSTE-ACS, and STEMI who were admitted to medical departments for cardiac catherization was lower during the lockdown period in 2020 than in the same non-pandemic period in 2019 [7, 9, 15, 17–19]. Given the potentially heightened environmental and psychosocial stressors and the fact that COVID-19 infections may induce acute cardiac injury or myopericarditis mimicking ACS, at least an increase in patients with ACS could have been expected [2–4, 13].

Potential reasons for the deferrals of these patients during the pandemic are different: First, there is a particular patient-based anxiety to come to the hospital due to COVID-19. This behavior was potentially reinforced by the official governments order to “stay at home”. Second, exhausted outpatient care capacities during lockdown might further delay cardiac care. Third, the triage of patients according to priority levels, with patients with assumed elective procedures being put on a waiting list, has kept patients from urgently seeking medical attention for chest pain [9].

When comparing in-hospital mortality in patients admitted for cardiac catheterization there was an increase in 2020 indicating that these patients were potentially referred too late to the hospital. This finding is in line with several reports in the literature demonstrating complications after myocardial infarction more frequently due to delayed presentation [20]. It has to be concluded that the fear of contracting COVID-19, although justified, here may have resulted in an increase in non-COVID morbidity and mortality caused by avoidance of the medical system.

In contrast to the higher rate of cardiovascular deaths, the rate of mortality following stroke decreased during the lockdown period. This finding is may potentially be explained by misdiagnosed or underdiagnosed stroke during lockdown. Accordingly, Rinkel et al. observed a 24% decrease in suspected stroke presentation during the lockdown period in the Netherlands [23]. The decrease in numbers of death due to other reasons during the lockdown period in 2020 compared with the non-pandemic period in 2019 might be explained by the fact that during the lockdown period there were fewer traffic accidents [24] and less crime [25].

Catheterization activities from the high-volume centers in central Germany were similar in the pre-pandemic when compared to 2019 excluding any influence of recently published studies (e.g. ISCHEMIA Trial) regarding the optimal indication for coronary angiography. However, in the present study, possible changes in local protocols of ACS patient management during the COVID-19 pandemic were not assessed. Since the proportion of complex cardiac catherization procedures did not increase during the pandemic-related lockdown, a balanced and appropriate prioritization of elective procedures in the hospitals can be assumed. Most importantly, the adequate care of STEMI patients in line with the guidelines was maintained despite potential locally limited resources, as evidenced by similar door-to-balloon times.

An inherent limitation of the present study is the lack of an autopsy-based diagnosis confirming the definite cause of death. Causes of death were documented by a physician at the last medical contact. This could mean that an existing COVID-19 diagnosis wrongly failed to state other causes of death. Moreover, COVID-19 infections could also have been overlooked due to the fact that thromboembolic or atherothrombotic events leading to death and listed as the cause of death, e. g. myocardial infarction, could be at least partially related to an existing non-diagnosed COVID-19 infection. Nevertheless, this was performed consistently during the pandemic period as well as during the non-pandemic period in 2019. In the present study the proportion of patients who died in hospital or out of hospital was not assessed. Given this fact, unrecognized/undiagnosed COVID-19 deaths have to be considered. However, due to the extensive testing for COVID-19 in central Germany during the lockdown period we assume a negligible number of misdiagnosed patients; conversely, cases of sudden cardiac death may have been attributed erroneously to COVID-19. According to recently published data, stent thrombosis occurred significantly more frequently in patients with COVID-19 infections [26]. Additionally, a German COVID-19 autopsy study revealed a remarkable rate of deaths (5%) finally defined as non-COVID 19 deaths with virus-independent causes (e.g. pulmonary embolism or myocardial infarction).

A further potential limitation is that only patients who were admitted for cardiac catheterizations were included in the present study. Thus, admissions for other cardiovascular reasons (e.g. heart failure, arrhythmias, pulmonary embolism) during the pandemic-related lockdown were not assessed. However, it must be assumed that hospitalization for other cardiovascular reasons also declined,; e.g. Bromage et al. showed a significant decrease in acute heart failure admissions [6].

In summary, the common recommendation to defer elective cardiac procedures during the lockdown phase of the COVID-19 pandemic of 2020 in order to preserve resources (including personal protective equipment and hospital beds) led to a restrictive attitude in the use of the highly developed healthcare system in central Germany. This consequently led to reduced admissions for elective cardiac catherization and, even more critically, to a reduction of referrals for ACS.

It can only be speculated whether this restricted healthcare affected the observed increase in cardiovascular and cardiac mortality during the COVID-19 pandemic-related lockdown and the associated social distancing restrictions; however, national programs should be designed to counteract the fact that under such conditions patients at higher cardiovascular risk hesitate to seek cardiac care when symptoms occur. The data emphasize the importance of maintaining low-threshold access to cardiovascular care during such a pandemic.

## Data Availability

Yes.
